# Oral health inequality among Indonesian workers in South Korea: role of health insurance and discrimination factors

**DOI:** 10.1186/s12903-022-02050-3

**Published:** 2022-01-29

**Authors:** Herry Novrinda, Dong-Hun Han

**Affiliations:** 1 Department of Dental Public Health and Preventive Dentistry, Universitas Indonesia, Jakarta, Indonesia; 2 Department of Preventive and Social Dentistry, School of Dentistry, Seoul National University, Seoul, 08826 South Korea; 3 Dental Research Institute, Seoul National University, Seoul, South Korea

**Keywords:** Disparities, Migrant worker, Discrimination, Indonesia, South Korea

## Abstract

**Background:**

The health of migrant workers is becoming an important public health issue. Although there are an increasing number of migrant workers in Korea, the health status in migrant populations remains unknown. The aims of this study were (1) to evaluate the association between income and self-rated oral health (SROH), and (2) to assess the role of health insurance and self-perceived discrimination in the association between income and SROH among Indonesian migrant workers in Korea.

**Methods:**

Information about self-reported income, SROH, coverage/utilization of health insurance (HI), living difficulties related to oral health (LDROH), oral health literacy (OHL), and discrimination were obtained from Indonesian migrant workers in Korea (n = 248). The main explanatory variable was income, and SROH was an outcome variable. Logistic regression analyses were performed controlling for age, gender, HI, LDROH, OHL, and discrimination. The paths from income to SROH were analyzed using the Partial Least Square-Structural Equation Model (PLS-SEM).

**Results:**

Among Indonesian migrant workers, the lower income group had the highest probability of a poor SROH compared to the higher income group. The variables showing a high explanatory power were discrimination among the low income group and HI among the middle income group. In PLS-SEM, the variables such as HI, LDROH, OHL, and discrimination contributed 11% to explaining the association between income and SROH.

**Conclusion:**

A monotonic gradient was revealed among migrant workers according to the association between income and SROH. Discrimination and HI contributed to oral health inequalities.

## Background

World trade, characterized by the movement of goods, services and people, is one of the important developments of the modern global economy. In particular, a sharp increase in workforce mobility has been observed in Asian countries [1]. For example, South Korea is one of the main destination countries for migrant workers from various Asian countries. In 2019, there were more than 2.36 million foreigners in Korea. Among them, more than 0.28 million foreigners were ‘Non-professional employment (E-9 Visa),’ and they entered Korea mainly to solve the labor shortage in low-wage ‘dirty, dangerous, and demeaning profession’ often performed by blue-collar workers. As for the nationalities of the migrant workers with the status of ‘Non-professional employment (E-9 Visa),’ Cambodia occupied the largest share at 13.7% followed by Vietnam (13.5%), Nepal (13.0%), Indonesia (10.3%) and Thailand (9.5%) [2, 3].

Migrant workers as part of the labor force might be a vulnerable group because they are foreigners working in a poor workplace environment with low-wages [4]. Previous studies on the health of migrant workers from abroad have shown that relationship with family and neighborhood [5], health service seeking behavior [6] and experience of discrimination [7] were associated with self-rated oral health (SROH). Other studies on migrant workers in various countries found that their barriers to health-care access are attributed to their lack of familiarity with the health-care system, language barriers [8] and lack of awareness of their rights and entitlements to health care as provided by their medical insurance [9].

There is growing evidence that perceived discrimination is associated with lower levels of physical and mental health, poor access to quality health care, and certain health behaviors across several immigrant groups [10]. Migrant workers in Korea have faced problems such as hazardous occupational conditions, low wages, long working hours, language barriers, lack of knowledge about the utilization of Korean health services, time constraints, and so on [11].

Unlike in Western industrial countries, the health status of migrant workers in Korea has not been studied well. Moreover, there are only a few studies on migrant workers regarding their oral health conditions in Korea [12]. For instance, Choi et al*.* explained dental health-seeking behavior among migrant workers (non-Indonesian) in *Pocheon City* [12]. Accordong to the study, the prevalence of unmet dental need was 44%. The main reason of unmet dental need was time (55.2%) and dental expenses (11.9%). To the best of our knowledge, there are no studies on the social determinants of oral health among the migrant workers in Korea so far. Moreover, most of studies are dealing with health inequality of immigrants compared to natives. Rare study has been done about health inequality among immigrants. Only one recent study which investigated the association between healthcare needs and socioeconomic status among immigrant women has been found [13]. According to the previous study, the main barrier to the immigrant women's healthcare needs was "financial pressure" and health inequality among aged immigrant women was due to economic factors. This type of research might enlighten our insight into the inequality of immigrants in multicultural society.

To suggest potential factors explaining the inequality in SROH among migrant workers in South Korea, various social determinants have been examined in a previous studies including health insurance (HI), living difficulties related oral health (LDROH), oral health literacy (OHL), and discrimination [14–19]. From the findings of previous study [13], we derived the following concept explaining the complexity of oral health inequality; The magnitude and perhaps the nature of social determinants such as HI, OHL, and discrimination which affect migrants vary by their income level. Therefore, we made a conceptual framework in explaining the inequality in the SROH with the above mentioned social determinants (HI, LDROH, OHL, and discrimination), tested its significance, and finally constructed an analytical model to clarify the inequality in the SROH through above mentioned social determinants.

The hypotheses of this study were two-fold: there is a social inequality in the SROH, and the inequality of the SROH according to income might be mediated by socioeconomic factors and self-perceived discrimination among Indonesian migrant workers in Korea. Accordingly, the overarching objectives of this study were (1) to assess the relationship between income and SROH, and (2) to assess the role of HI, LDROH, OHL, and self-perceived discrimination in the association between income and SROH among Indonesian migrant workers.

## Materials and methods

This was a cross-sectional study to find the role of HI, LDROH, OHL, and self-perceived discrimination in the association between income and SROH among Indonesian migrant workers. It was approved by the Institutional Review Board of the Seoul National University School of Dentistry (No.S-D20180027). Written informed consent was obtained from all participants.

### Participants

The study participants were recruited in cooperation with several Islamic religious communities. We thought that 20 Indonesian migrant workers from each level of income (3 levels), HI (2 levels) and discrimination (2 levels) should be analyzed. Therefore, we had planned to meet with at least 240 Indonesian migrant workers and ended up recruiting more to take into consideration those who refused to respond to the survey. A total of 256 participants were recruited from November 2018 to January 2019, and the purpose and method of this study were explained to them. The participants were selected by a snowball sampling method from Islamic religious communities. This method was used because Indonesian migrant workers gather and worship at Muslim religious communities every Friday. All the participants voluntarily responded to the survey (response rate 100%), agreed to join this study and gave their written informed consent. The survey was conducted at the Islamic mosques where the study participants could respond to the survey in a familiar and comfortable manner. The criteria for inclusion and exclusion were as follows.

- Inclusion criteria

· those aged 20 years or older Indonesian migrant workers

· those willing to participate in this study

- Exclusion Criteria

· those who cannot understand and respond to the questionnaire by themselves

· those who no longer want to participate in the research during the course of the study

In the case of questionnaires with missing values, 8 cases were deleted, and the final sample size for the analyses was 248.

### Questionnaire

A preliminary study was conducted to construct the questionnaire. A group discussion regarding relevant issues was held with six Indonesian migrant workers. A list of open questions such as demographics, coverage and utilization of health insurance, complaints regarding dental and oral health, daily life such as access to places of worship and *halal* food (for Muslims), and experiences of discrimination when they were in Korea were arranged through dialogue and discussion. According to these dialogue and discussion, we constructed a questionnaire including demographic features, income, occupation, education, coverage and utilization of health insurance, oral health, and self-perceived discrimination. A set of modified questionnaires were added to get the necessary variables based on references such as Health Literacy in Dentistry (HeLD) [20] and its translated version in Indonesian [21] and a questionnaire on living difficulties for immigrants (LDROH). After that, the final version of questionnaire was made.

Income was used as the independent variable because there is a great deal of homogeneity in other social status indicators such as occupation and education level. The majority of Indonesian migrant workers have a high school education level and work as non-professional workers. Self-reported income was obtained from an interview. The income was categorized into three groups: less than 1,750 USD, lower income; 1750–2200 USD, middle income and more than 2,200 USD, high income.

The dependent variable was the SROH. The SROH was widely used due to its convenience and reliability [22]. Indonesian migrant workers were asked the following by the SROH: “Overall, how would you rate your oral health?” and the responses were “very good, good, moderate, poor, or very poor.” Then, the SROH scale was converted into a dichotomous scale (very good to moderate as a good oral health and poor to very poor as a poor oral health).

HI consisted of coverage and utilization of health insurance. The coverage and utilization were coded as yes or no, respectively. LDROH consisted of 3 questions: 1) “Did you have communication difficulties or language barriers?” 2) “Did you have difficulties in understanding health / dental health information?” and 3) “Did you have poor access to dental care?” The responses were “yes or no.” OHL consisted of Korean proficiency and HeLD. Korean proficiency score was categorized by low (≤ 4) or high (≥ 5).” The HeLD consists of 29 items which cover seven oral health literacy domains: communication, understanding, receptivity, utilisation, support, financial and access. For each of the 29 HeLD questions, the responses were ‘difficulty’ experience or no experience. The HeLD score was categorized by less (≤ 6) or more (≥ 7). The participants were also asked about their self-perceived discrimination: “Since your arrival in Korea, have you experienced discrimination or been treated unfairly by others because of your ethnicity, culture, race, or skin color, language or accent, or religion?” and the responses were “yes or no.” It was used in previous studies in Canada [23], Sweden [24], and Australia [25].

### Statistical analyses

Data were analyzed using *SPSS ver 23.0*. Chi-square test for categorical variables was used to assess the associations of the outcome variable with the other variables. Independent t-test was performed to assess the difference in several continuous variables between the outcome groups. Odds ratios (ORs) of poor oral health were calculated according to the income level. Serial adjustment was applied for age (continuous), gender, HI, LDROH, OHL, and discrimination using logistic regression analyses. This strategy has been used previously to assess the contributing roles of potential mediating factors in oral health inequality [26, 27]. The reference group was high income. The baseline model (model 1) was adjusted for age and gender. The Partial Least Square-Statistical Equation Model (PLS-SEM) [28] with WarpPLS 7.0 [29] was also used. A model was setup to assess the direct and indirect effects of the income of Indonesia migrant workers in Korea on their oral health. The hypothesis was that the income of Indonesia migrant workers in Korea affects their SROH directly and indirectly by their OHL, HI, LDROH and self-perceived discrimination experience.

Considering several circumstances such as dichotomous variables, number of respondents, and the possibility of non-normal distribution of data, path coefficients of PLS-SEM were estimated by the asymptotic distribution free method (ADF). The PLS-SEM applied with ADF could be used for data that are nominal, ordinal, interval, as well as ratio and do not have a specific distribution pattern. Model fit was evaluated with proper indices such as the Average Path Coefficient (APC), the Average block VIF (AVIF), the Average Full collinearity VIF (AFVIF), the Tenenhaus GoF (GoF), Sympson's Paradox Ratio (SPR), R-Squared Contribution Ratio (RSCR), Statistical Suppression Ratio (SSR) and Non-Linear Bivariate Causality Direction Ratio (NLBCDR).

## Results

The final number of participants was 248 Indonesians consisting of various ages, genders, educational backgrounds, and other socio-demographic information. Among the Indonesian workers, the mean of age was 27.75 ± 5.86 years (male 27.79 ± 5.66 and female 27.06 ± 7.52 years). Male, high income, health insurance beneficiary, and those who have used health insurance were dominant over their counterparts. Age, gender, income, experience of health insurance usage, poor access to dental care, Korean proficiency score and discrimination experience showed no difference between SROH status. However, health insurance coverage, language barriers, understanding oral health information and HeLD score were associated with the SROH shown in Table 1.

**Table 1:** Characteristics of Indonesian migrant workers in Korea according to self-rated oral health

Variables		Self-Rated Oral Health (SROH)		
		Good, n (%)	Poor, n (%)	p^*^
Age, mean (standard deviation)		27.75 (5.86)	26.25 (7.57)	0.481^**^
Gender	Male	209 (87.1)	8 (100.0)	0.277
	Female	31 (12.9)	0 (0.0)	
Income	Low	28 (11.7)	2 (25.0)	0.519
	Middle	97 (40.6)	3 (37.5)	
	High	114 (47.7)	3 (37.5)	
Health insurance coverage	Yes	213 (88.8)	4 (50.0)	**0.001**
	No	27 (11.3)	4 (50.0)	
Health insurance use	Yes	108 (46.2)	2 (28.6)	0.357
	No	126 (53.8)	5 (71.4)	
LDROH				
Language Barriers	No	170 (70.8)	2 (25.0)	**0.006**
	Yes	70 (29.2)	6 (75.0)	
Understanding oral health information	No	133 (55.4)	1 (12.5)	**0.017**
	Yes	107 (44.6)	7 (87.5)	
Poor access to dental care	No	216 (90.0)	6 (75.0)	0.173
	Yes	24 (10.0)	2 (25.0)	
Oral health literacy				
Korean proficiency score	Low	71 (29.6)	4 (50.0)	0.216
	High	169 (70.4)	4 (50.0)	
HeLD score	Less	29 (12.2)	3 (42.9)	**0.018**
	More	208 (87.8)	4 (57.1)	
Discrimination experience	No	206 (94.5)	7 (87.5)	0.404
	Yes	12 (5.5)	1 (12.5)	

LDROH: Living difficulties related to oral health

HeLD: Health literacy in dentistry

Bold denotes statistical significant at p < 0.05

*Obtained from Chi-Square test (for categorical variables)

**Obtained from independent t-test (for continuous variable)

In an adjusted logistic regression analysis controlling for age and gender, the low-income group showed 2.16 odds of poor SROH (Model 1 in Table 2, 95% CI 0.82–5.68). This association became significant and strengthened to 2.97 in model 5 of Table 2 (95% CI 1.07–8.23). The *Δ*OR in model 2 to 5 explained the explanatory power of HI, LDROH, OHL and discrimination in the association between income and poor SROH. The *Δ*OR was calculated as [(OR_*model x*_—OR_*model 1*_) / (OR_*model 1*_ – 1)]*100%. The discrimination factor had the highest explanatory power of 69.57% (Model 5 in Table 2).

**Table 2:** Explanatory power of potential mediating factors in the association between income and Poor SROH among Indonesian Migrant Workers in Korea

		Model 1 OR (95% CI)	Model 2 OR (95% CI)	Δ OR %	Model 3 OR (95% CI)	Δ OR %	Model 4 OR (95% CI)	Δ OR %	Model 5 OR (95% CI)	Δ OR %
Income	High	1 (reference)	1 (reference)		1 (reference)		1 (reference)		1 (reference)	
	Middle	1.79 (0.89–3.60)	1.60 (0.77–3.33)	23.44	1.70 (0.82–3.50)	10.83	1.81 (0.90–3.66)	3.18	1.76 (0.83–3.75)	3.06
	Low	2.16 (0.82–5.68)	1.68 (0.59–4.76)	41.72	2.18 (0.79–5.99)	1.72	1.50 (0.51–4.39)	56.90	**2.97 (1.07–8.23)**	69.57

Model 1 was adjusted for age and gender

Model 2 was adjusted for age, gender (model 1) plus Health Insurance (HI)

Model 3 was adjusted for age, gender (model 1) plus Living Difficulties Related Oral Health (LDROH)

Model 4 was adjusted for age, gender (model 1) plus Oral Health Literacy (OHL)

Model 5 was adjusted for age, gender (model 1) plus Discrimination

ΔOR = ((ORmodel x—ORmodel 1) / (ORmodel 1 – 1))*100%

Bold denotes statistical significance at p < 0.05

**Fig. 1:**
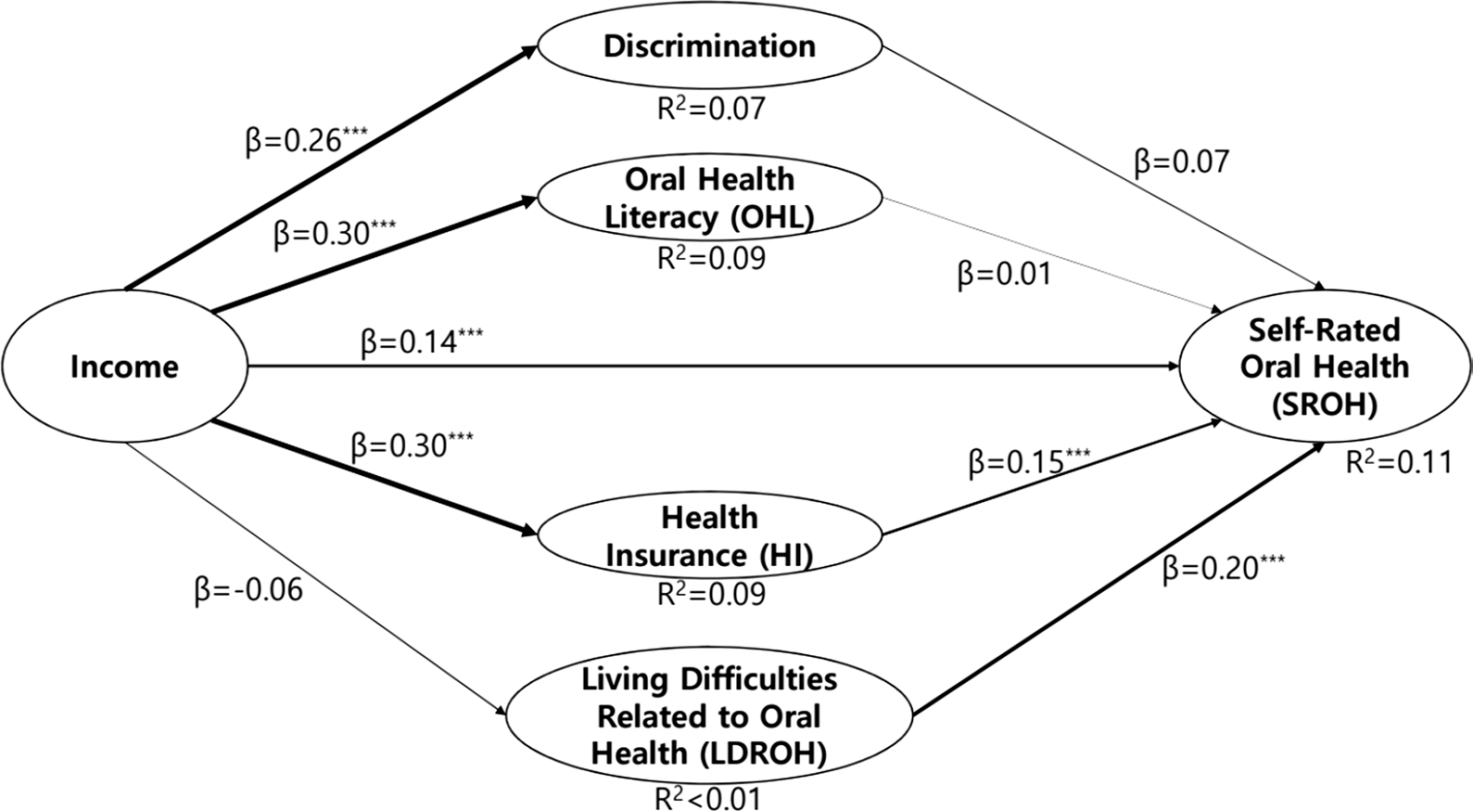
Path diagram of discrimination, oral health literacy, health insurance and living difficulties related to oral health as mediating factors in the association between income and self-rated oral health among Indonesian migrant workers

The path model of direct and indirect effects was evaluated between the variables of interest such as income, OHL, LDROH, HI, discrimination and SROH. PLS-SEM was performed, and Fig. 1 shows that for direct effect of income was significant, in other words, the direct effect of income was supported (β = − 0.14). Indirect effect between income and SROH which was mediated by HI, was also significant (income to HI: β = 0.30; HI to SROH: β = 0.15). The total indirect effect was calculated from the sum of the income-(discrimination, HI, OHL, LDROH)-SROH pathways; (0.26 * 0.07) + (0.30 * 0.01) + (0.30 * 0.15) + (− 0.06 * 0.20) = 0.22. Then, the total effect was resulted from the sum of the direct and indirect pathways: 0.14 + 0.22 = 0.36 [28].

Thus the income of Indonesia migrant workers in Korea was directly associated with SROH, and income was also indirectly associated with the SROH through HI but not through OHL, LDROH, and self-perceived discrimination (Fig. 1). The fit of the path model was assessed using classic and additional indices. For the classic indices, APC was 0.082 (*p* = 0.047); AVIF was 1.009, and AFVIF was 1.024 (the values of AVIF and AFVIF are regarded as ideal if ≤ 3.3); GoF was 0.120 (the value was regarded as small). For the additional indices, SPR was 0.889 (the value was regarded as acceptable if ≥ 0.7, ideally = 1), RSCR was 0.997 (the value was regarded as acceptable if ≥ 0.9, ideally = 1), SSR was 1.000 (the value was regarded as acceptable if ≥ 0.7) and NLBCDR was 0.778 (the value was regarded as acceptable if ≥ 0.7). All the indices were above the recommended criteria for good of fit / acceptable fit [29]. The R^2^ was 0.11 (11%).

## Discussion

Factors reflecting the socioeconomic position (SEP) had a negligible association with the SROH. This finding differed slightly from other studies that found a relationship between the SEP and SROH [19, 30, 31]. Hakeberg et al*.* [30] reported this finding in Swedish adults, Tsuboya et al*.* [19] in Japanese workers, and Guarmizo-Herreno et al*.* [31] in the adult population of England, Wales, and Northern Ireland. The difference might be attributed to the small number of migrant workers, whereas the other studies were national surveys with thousands of participants.

Health insurance coverage, language barriers, understanding oral health information and health literacy in dentistry score were all associated with SROH in a significant way. These findings add to the findings of other studies from various countries. The SROH was found to be significantly associated with clinical findings and unmet treatment needs [32–34] and with general health and daily function in older adults [35] [1–3] ^1–3^. The findings should be considered as insight into future studies and/or policies because the aforementioned factors are also associated with the SROH. Business owners paid 40.8 percent of the medical costs for injuries among foreign workers; 29.3 percent was paid by Industrial Accident Compensation Insurance and 29.9 percent was paid by others [36]. Such data may imply that most foreign workers were not covered by the National Health Insurance (NHI) in Korea, even though one of the main barriers to access health care by migrant workers is insurance coverage for medical expenses [37]. Health insurance and language barriers should be given more serious consideration by policymakers from both parties. Due to the unavailability of health insurance in employment permit system (EPS) schemes [38], one of the top priorities is to ensure that health insurance is included in EPS.

Another way to overcome such barriers is to extend the time for Korean language training, which will be accompanied by additional health lessons such as oral health and personal hygiene. Given that the cost for the Test of Proficiency in Korean (TOPIK) for prospective migrant workers is quite high (approximately US$1500), which is five times the average monthly salary in Indonesia [39], it is well worth the effort to incorporate current (oral) health related issues into their training. This kind of additional information is very urgent considering the Covid-19 global pandemic and its spread in both Korea and Indonesia. The authorities must ensure that prospective Indonesian migrant workers have a thorough understanding of health (including oral health) issues. Simultaneously, Indonesian oral health professionals will be able to contribute by participating in the process of sharing information about health issues (including oral health) for migrant workers in Indonesia while Korean oral health professionals may be able to contribute by providing health information that is easily understood by foreigners, particularly Indonesians.

A study in Iran showed that a low oral health literacy level, independent of education and other socioeconomic determinants, might be a predictor for poor self-reported oral health and be considered as a vital determinant of oral health in countries with developing health-care systems [17]. Length of stay (time since immigration) could also be related to oral health as a finding in Ontario [14]. Oral health literacy occurs within diverse frameworks of cultures and economies, education systems and individual health system experiences. A thorough understanding of oral health literacy can contribute to and complement health, oral health, health outcomes and costs [40].

Migrant workers come from Indonesia, one of the developing countries in Southeast Asia, so even though they work in Korea, an advanced industrial country, demographic characteristics such as the level of education and social strata in the area of origin remain attached to migrant workers. Language and cultural differences may also contribute to their deteriorating oral health. Language barrier is associated significantly with the SROH. Although they have passed the TOPIK as one of the requirements to be able to work in Korea [38], this language proficiency is more geared towards the needs of daily life while the terminology used in the field of (oral) health such as in pamphlets about health and dental care or other oral health promotion media, are still less familiar. These factors, along with several other factors as listed in the health literacy skills (HLS) framework [41] have been hypothesized to have an impact on health literacy skill. Therefore, identifying these factors is expected to provide guidance for developing policies and programs to improve oral health literacy among Indonesian migrant workers.

The low SEP had the highest likelihood of a poor SROH. Despite differences in sample size and coverage of participants, this finding was consistent with several previous studies. A Lower SEP was also associated with a poorer SROH among adults in England [42] whereas adults in Southern Brazil with a ‘decreased income’ were more likely to have a poor SROH [43]. The lowest income group in Canada was more likely to have a poor SROH than the highest income group [44]. According to the data from the Indonesian National Socio Economic Survey (*Susenas*), the richest groups (quintile 4 and 5) have the lowest perceived need for dental care [45]. The SEP, as reflected by the income indicator, continues to show health inequalities, even among migrant workers. By considering the vulnerabilities of migrant workers, we might be able to determine the magnitude of inequality between Korean and migrant workers. This assumption should serve as a trigger for all parties to make concerted efforts to improve the (oral) health of migrant workers to reduce the gap.

The discrimination factor was significant for a poorer SROH status among lower-income Indonesian migrant workers. This should be a serious concern for interested parties because discrimination is not only limited to migrant workers. It also affects Koreans and has a negative impact on their health. Gender and education levels are the most common sources of self-reported discriminatory experience, according to previous studies, and there is a dose–response relationship between the number of situations of discriminatory experience and a poor SRH. The lowest income group also had the highest proportion of perceived discrimination and the lowest poor SRH [46, 47]. Workplace discrimination was positively related to psychological distress in a sample of unskilled Indonesian migrant workers in Malaysia [48]. Similar to these findings, previous studies have shown that discrimination has hampered access to medical and dental care for a variety of groups, including immigrants and indigenous people [49, 50]. Among certain US populations, perceived discrimination was related to income and race. Individuals with medium–low incomes were significantly more likely to face discrimination than those with high-incomes [51]. The absence of dental visits was significantly associated with self-reported discrimination among Aboriginal Australians [25].

The statistical analyses (logistic regression and SEM) revealed that health insurance and discrimination are relevant factors accounting for a portion of the association between SEP and oral health. This finding extends previous studies on the role of health insurance [52–55] and discrimination [24, 25, 46–49, 51, 56] as well as explaining (oral) health inequalities across countries.

These overall findings support the assertion that discrimination affects many social groups, particularly visible minorities [49]. Therefore, the assumption that the absence of health insurance in the EPS constitutes discrimination against migrant workers cannot be completely dismissed. Minimizing discrimination, particularly in the utilization of (oral) health services, appears to reduce oral health inequalities among migrant workers in Korea. Discrimination that jeopardizes access to care is morally unacceptable and against the universal values of equality, justice, social solidarity, and the public health perspective [49].

The overarching findings of this study should be interpreted taking into consideration some of its limitations. First, the design was a cross-sectional study, so the direction of the causality relationship could not be described. Second, due to a variety of factors, the sample size of migrant workers was small, limiting the scope of the analysis. Third, although self-reported oral health condition is considered reliable [22], subjectivity in the responses of the respondents is very likely to occur considering that the variables were collected based on self-reported conditions. Fourth, this study focused on migrants from Indonesia. The biggest reason for conducting this study on Indonesian immigrant workers was that it was easier in terms of culture and language to conduct the study on the author's homeland workers. If the study had been conducted on immigrant workers from various countries, the results of the study could have been generalized. However, there must have been difficulties in reliability and standardization due to different cultures and languages. Fifth, all the participants in our study were Muslims. By focusing on the Islamic religious communities, we might miss to recruit representative Indonesians. However, because Indonesia is the world’s largest Muslim country, with about 88% of the population being Muslim, the participants in our study could represent Indonesian migrant workers. In addition to its limitations, this study may be regarded as the first piece of research on the oral health of Indonesian migrant workers in Korea. It is also the first model to introduce the role of discrimination and health insurance in oral health inequalities among Indonesian migrant workers and providing future research direction on the oral health of migrant workers.

## Conclusion and suggestion

A monotonic gradient was revealed among migrant workers according to a pragmatic approach (single / income indicator). Discrimination factor was the highest explanatory power for all social class positions and contributed the most to oral health inequalities and was significant for the lower income group among Indonesian migrant workers. Discrimination and health insurance factors had a significant role in explaining the association between SEP and oral health inequalities among them.

Minimizing or eliminating opportunities for discrimination against all workers need to be enforced, particularly for disadvantaged groups such as low-income migrant workers. Migrant workers should become an inseparable part of the Korean workforce by integrating them through policies that are on par with the policies for Koreans. Related parties in both Korea and Indonesia should improve the welfare of migrant workers including ensuring access to dental care and other oral health efforts through integrated programs. All these recommendations are expected to reduce social inequalities related to oral health in Korea. We hope that this study will increase public awareness and be considered by policymakers and stakeholders in both Indonesia and South Korea to improve the health of migrant workers.

## Data Availability

The datasets used and/or analysed during the current study are available from the corresponding author on reasonable request.

## Abbreviations

AVIF: Average block VIF
AFVIF: Average full collinearity VIF
APC: Average path coefficient
GoF: Tenenhaus GoF
HI: Health insurance
HeLD: Health literacy in dentistry
LDROH: Living difficulties related to oral health
NHI: National Health Insurance
NLBCDR: Non-Linear Bivariate Causality Direction Ratio
OHL: Oral health literacy
PLS-SEM: Partial Least Square-Statistical Equation Model
RSCR: R-Squared Contribution Ratio
SPR: Sympson's Paradox Ratio
SROH: Self-rated oral health
SSR: Statistical Suppression Ratio
STDCR: Standardized Threshold Difference Count Ratio
TOPIK: Test of Proficiency in Korean
